# The Mosquito Pest

**Published:** 1908-05

**Authors:** 


### THE MOSQUITO PEST.

It will certainly be an unwise policy for the city authorities of Atlanta to allow the mosquitoes to become unnecessarily numerous this summer on account of the shrinkage of the city's income. The curtailment of appropriations should not affect the $5,000 necessary to annihilate the mosquitoes by attacking their larvae. This may be done by coating the water with crude oil. It is hoped that when Dr. J. P. Kennedy explains the situation to the Finance Committee that they will see the urgent need of this appropriation to carry on the war against the mosquito.

